# Two Cases of Mycotic Aneurysms Caused by *Brucella suis* Infection of Aortic Graft Material

**DOI:** 10.1093/ofid/ofaf404

**Published:** 2025-07-09

**Authors:** Brandi M Mize, Emily Laskey, Gregory L Damhorst, Colleen S Kraft, Guillermo A Escobar

**Affiliations:** Division of Vascular Surgery and Endovascular Therapy, Department of Surgery, School of Medicine, Emory University, Atlanta, Georgia, USA; School of Medicine, Emory University, Atlanta, Georgia, USA; Division of Infectious Diseases, Department of Medicine, School of Medicine, Emory University, Atlanta, Georgia, USA; Department of Pathology and Laboratory Medicine, School of Medicine, Emory University, Atlanta, Georgia, USA; Division of Vascular Surgery and Endovascular Therapy, Department of Surgery, School of Medicine, Emory University, Atlanta, Georgia, USA

**Keywords:** aortic brucellosis, *Brucella*, mycotic aortic aneurysm, vascular surgery

## Abstract

*Brucella suis*, a zoonotic pathogen, can affect multiple human organ systems causing various clinical manifestations. While aortoiliac involvement is rare worldwide, we report 2 cases of aortic brucellosis following abdominal aortic aneurysm repairs within a 9-year period at a single US institution in Georgia. One case was an infected aortic endograft, which may be the first reported. *B suis* aortoiliac infections are rare even in endemic areas, thus highlighting how uncommon these cases are in Georgia. Acknowledging the dismal prognosis with symptomatic aortic graft infections, we wish to share our experience in successfully treating them, including an infected aortic endograft. We recommend obtaining a robust history when evaluating individuals with suspected mycotic aneurysms who frequently handle animals. Education on protective equipment and proper handling of animals is imperative to reduce the risk of aortic graft brucellosis infections. Our institutional experience suggests that graft explantation and doxycycline-rifampin are acceptable treatment options.

Brucellosis is a systemic infection caused by bacteria of the genus *Brucella*, a zoonotic pathogen frequently encountered when handling infected livestock or wild game usually in endemic areas. Brucellosis can involve nearly all body systems but most often targets the musculoskeletal system, presenting as flu-like symptoms [1, 2]. Cardiovascular involvement affects approximately 3% of patients with brucellosis worldwide with a subset including aortoiliac involvement, an even rarer manifestation but with a staggering mortality rate ranging from 12.3% to 22% [1–4]. Aortoiliac brucellosis is often reported in endemic areas such as Mediterranean countries and China, with the United States, which is not an endemic area, accounting for only 11 of 130 cases reported in a large systematic review [3, 4]. Symptoms associated with aortoiliac brucellosis are generally nonspecific, presenting as chest, abdominal, or inguinal pain with fever and malaise, making the diagnosis difficult.

We report 2 cases of aortic brucellosis caused by *Brucella suis* at a single institution in the United States, 1 of which we believe is the first record of successfully treating a *Brucella*-infected endograft with a rifampin-soaked Dacron graft. Given the rare diagnosis of aortic brucellosis in the United States, we aim to add to the literature by including clinical presentation, diagnostic workup, and management to highlight these uncommon cases and their collaborative management.

## CASE 1

A 48-year-old male presented to our institution as a transfer in the summer of 2020 for a suspected symptomatic mycotic abdominal aortic aneurysm (AAA) with endograft involvement. Clinical suspicion was based on cross-sectional imaging and a 1-month history of fevers, chills, and malaise with anorexia and poorly controlled and intense lower back pain. Past medical history included a saccular AAA treated by endovascular aneurysm repair (EVAR) 4 months prior. His remaining pertinent medical history included congestive heart failure and a 3-vessel coronary artery bypass.

**Figure 1.**
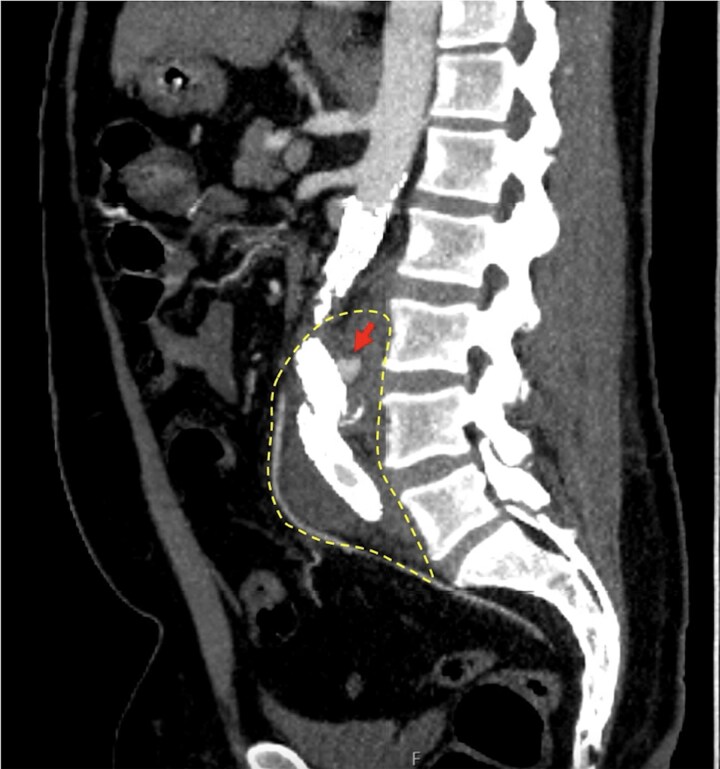
Case 1: sagittal cut of computed tomography angiogram demonstrates contrast extravasation (arrow) at the junction of the left iliac limb of the endovascular aneurysm repair, suggestive of a type III endoleak. Perigraft soft tissue stranding (dashed circle) is suggestive of phlegmon.

**Figure 2.**
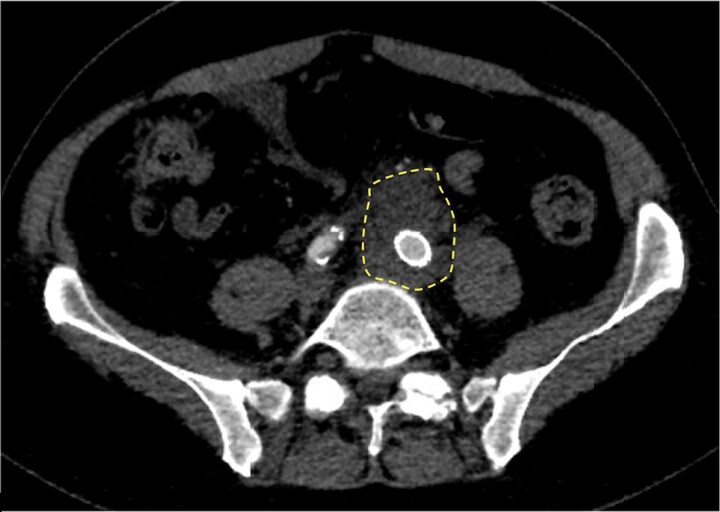
Case 1: axial cut of computed tomography angiogram shows soft tissue stranding around the left iliac limb of the endovascular aneurysm repair (dashed circle), suggestive of a thick phlegmon from a mycotic abdominal aortic aneurysm with an infected endograft.

He was hemodynamically stable on arrival to our facility. His examination was significant for a palpable pulsatile mass in the left lower abdominal quadrant with associated tenderness and radiating pain to his back. Upon review of the computed tomography angiogram (CTA) performed at the outside hospital, there was immediate concern for interval development of contrast extravasation from the aortic endograft into the aneurysm sac, suggestive of an endoleak at the junction of the left iliac limb of the EVAR (Figure 1) and a thick phlegmon concerning for progression to a mycotic AAA in this same area (Figure 2). An emergent endovascular relining with a new iliac limb stent was performed to seal the suspected endoleak, thereby relieving pressurization of the mycotic aneurysm, which was successful and thought to be the primary cause of his back pain.

After an attempt to identify a blood-borne organism and preoperative optimization, the patient underwent surgery for his mycotic AAA 11 days after the relining surgery. Open removal of the infected aortic endograft was performed with local debridement of the phlegmon and aortic reconstruction with an aortobiiliac bypass via a rifampin-soaked Dacron graft. On postoperative day 4, the intraoperative cultures grew *B suis*, identified by matrix-assisted laser desorption ionization–time of flight. Based on literature for antibiotic regimens for *Brucella* endocarditis [5 ], the patient received intravenous gentamicin for 4 weeks and a 12-week course of oral doxycycline (100 mg, twice per day) and oral rifampin (900 mg, daily) with plans to remain on lifelong suppressive doxycycline. On review of risks factors for *Brucella* infection, the patient endorsed regularly hunting deer and feral hogs, with his last exposure to a feral hog being 1 month prior to his index EVAR and 5 months prior to this current presentation. While he did not consume or process the meat himself, he did field dress the feral hogs, which entailed direct contact with the animal's blood and internal organs. We believe that this case is the only known *Brucella* infection of an aortic endograft successfully treated with a rifampin-soaked Dacron graft.

Approximately 7 weeks after discharge, the patient followed up with his vascular surgeon and expressed an increase in appetite as compared to pre-surgery. He had stopped gentamicin and rifampin due to nephrotoxicity and associated gastrointestinal symptoms (ie, nausea and vomiting), respectively. He remained asymptomatic with doxycycline alone at that time. CTA of the abdomen and pelvis showed a patent graft reconstruction without obvious perigraft fluid collections or phlegmonous changes. At his 3-month follow-up, he remained asymptomatic with repeat CTA having no specific concerns for infection. He endorsed chills that coincided with cessation of his doxycycline, so the patient was advised to maintain lifelong suppressive doxycycline. On last contact with the patient in early 2025, he follows with an infectious disease specialist, is compliant with his doxycycline, and remains asymptomatic.

## CASE 2

A 70-year-old male with a history of a symptomatic inflammatory infrarenal AAA status after open repair with a Dacron graft 15 years ago presented to our institution in the fall of 2011 as a transfer from an outside hospital given concerns for an aortoenteric fistula. His symptoms included left-sided back pain and bright red blood per rectum. He endorsed a several-month history of intermittent melenic stools and constipation and a 4-year history of the left-sided back pain. His remaining pertinent medical history included coronary artery disease with previous myocardial infarction, peripheral artery disease, and hypertension. He was an active smoker and disclosed a 6-month history of anorexia with unintentional 40-lb weight loss.

On arrival, he was hemodynamically stable with leukocytosis and intact palpable femoral and pedal pulses. CTA was concerning for a mycotic AAA with foci of air in the aneurysm sac adjacent to the bowel and colon as well as an inflamed left psoas muscle. He underwent a staged procedure, with the first stage involving creation of an extra-anatomic axillary-bifemoral bypass to perfuse his bilateral lower extremities. The second stage was performed the following day, starting with the patient undergoing an exploratory laparotomy. A large phlegmon was exposed in the retroperitoneum with adhesions in the area that appeared to be related to chronic diverticulitis. Given the concern for chronic diverticulitis and a possible aortoenteric fistula, a general surgeon performed a sigmoidectomy with an end colostomy and Hartman pouch. After obtaining proximal and distal control of the aorta and common iliac arteries, the aneurysm sac was incised and purulence was encountered. The Dacron graft from the AAA repair 15 years prior was explanted. The infrarenal aorta and common iliac arteries were suture ligated.

On postoperative day 3, intraoperative cultures grew *Bacteroides theaiotamicron*, so the patient was treated with vancomycin and piperacillin-tazobactam and then transitioned to metronidazole on postoperative day 18. On postoperative day 14, intraoperative cultures grew *B suis* for which doxycycline and rifampin were initiated. He took rifampin and doxycycline for a total of 6 weeks. Upon further interview, the patient endorsed a 3-year history of systemic symptoms of fever, weight loss, and malaise for which he visited several doctors and no diagnosis was made. On review of risk factors for *Brucella* infection, he disclosed owning a goat farm, consuming unpasteurized raw goat milk for the past 4 years, and having field dressed a feral hog. The patient's postoperative course was complicated by myocardial infarction with ventricular fibrillation arrest, serial bedside midline abdominal wall debridements, and gastrostomy tube placement for adequate caloric intake. Unfortunately, the patient died approximately 3 months after the index procedure for this admission.

## DISCUSSION

Aortoiliac brucellosis is rare in nonendemic countries and can result in a variety of clinical manifestations, which commonly include fever and arthralgias [1, 2]. Nonspecific symptoms allow the infection to remain undetected for years, as highlighted in our second case. Supporting current literature, our patients presented with constitutional symptoms of fever, malaise, and unintentional weight loss [4, 6]. When coupling these symptoms with new-onset abdominal, back, flank, or inguinal pain in a patient with an aortic aneurysm or history of aneurysm repair, there should be a high clinical suspicion for development of a mycotic aneurysm. While the most common pathogens causing mycotic AAAs are *Salmonella* species (33%), *Staphylococcus* species (15.6%), and *Streptococcus* species (10.4%), *Brucella* can infect healthy aortic tissue and cause mycotic aneurysmal degeneration due to seeding of the pathogen in the vessel wall [7]. Likewise, *Brucella* can infect damaged tissue or invade prosthetic material and accelerate preexisting aneurysmal degeneration, as seen in these 2 cases. Similar to other literature, both patients presented with involvement of the abdominal aorta, which is the most common vessel affected by mycotic aneurysms caused by *Brucella* species [3, 4, 7, 8]. Additionally, our cases involved infection of existing aortic grafts from prior AAA repairs. We are unaware of any reports describing an infected aortic endograft by *Brucella*, thus highlighting the novelty of case 1, despite endografts becoming more commonly used to treat AAA and unstable primary aneurysms in patients with aortic brucellosis [3, 4, 9].

Human brucellosis is frequently associated with consumption of unpasteurized dairy products and contact with feral animals [10]. Here, we present 2 patients most likely infected by their interactions with feral hogs. Interestingly, both patients presented with mycotic aneurysms caused by *B suis*, a porcine pathogenic strain with high virulence, which is a rare manifestation of the disease and suggests that feral swine were the likely source in both cases [11]. While being an exceedingly rare cause of mycotic aneurysms in the United States, *B suis* infections are rare in areas where *Brucella* species are endemic. Cascio et al performed a systematic review of aortic brucellosis in which 24 of the 44 cases had *Brucella* species identified and only 3 of the 24 cases were caused by *B suis* [8]. In a more contemporary systematic review by Li et al, 44 of 130 aortoiliac brucellosis cases had *Brucella* species reported, with only 5 of the 44 cases being caused by *B suis* [4].

Both cases were managed with operative explantation of the infected aortic graft material and treatment with doxycycline and rifampin, an antibiotic regimen that has shown success in previous studies [4, 6]. In the first case, gentamicin was included as part of the regimen, while in the second case it was not. Although considered to have superior efficacy [12], inclusion of an aminoglycoside is often a dilemma for the managing team due to the high incidence of renal injury and ototoxicity and the requirement for intravenous administration, factors that likely underlie the difference in antimicrobial management in our cases. Here, even if the history of hog exposure were obtained before microbial identification, *Brucella* infections are sufficiently rare that an aminoglycoside-containing regimen would not have been included in empiric antimicrobial coverage given concerns that toxicities outweigh the potential benefit. Although the patient in our second case died, it is unclear if this was due to other factors or if it was a lack of antimicrobial efficacy.

Our first case highlights the utility of using a rifampin-soaked Dacron graft to lower the risk of recurrent aortic infection. Rifampin-soaked prosthetic grafts are a long-standing and effective practice in vascular surgery for procedures in infected or presumed infected fields, especially if no autologous vein conduit is available [13, 14]. While this was established in the 1990s, to our knowledge, this is the first description of utilizing a rifampin-soaked Dacron graft to treat a mycotic aneurysm specifically from *B suis*, which proved to be an effective addition in the treatment algorithm.

We reasoned that the prosthetic graft was a nidus for infection in both patients. The Society for Vascular Surgery guidelines for AAAs currently suggests antibiotic prophylaxis only for procedures with a high infection risk, such as dental procedures [15]. However, these cases make us wonder if patients with a high risk of exposure to *Brucella* species may benefit from counseling and/or prophylaxis to prevent complications of infected prosthetic graft material. In case 1, the exposure to *B suis* likely preceded the original EVAR placement, while in case 2 the exposure likely occurred after the open repair of the AAA 15 years prior. While the use of doxycycline (100 mg twice per day) and rifampicin (600 mg daily) for 21 days has been studied for preventing brucellosis in healthy individuals with occupational exposures, few studies have investigated the use of doxycycline-rifampicin as prophylaxis and treatment in patients with aortic graft placement and high-risk recreational exposures [16]. Therefore, it is important to consider the use of prophylactic antibiotics in patients for whom exposure to animals at risk for transmitting *Brucella* species is not a risk that they are able to modify in the postoperative period. Furthermore, it is important to (1) properly assess for these exposures in patients presenting with mycotic aortic aneurysms, (2) counsel patients regarding high-risk recreational exposures, and (3) provide guidance regarding the proper use of protective equipment and the proper disposal of animal carcasses as advised by the Centers for Disease Control and Prevention [17].

These cases highlight the importance of evaluating at-risk activities when a mycotic aneurysm is diagnosed. While no specific associations can be drawn between the medical and surgical management of our patients with *B suis* mycotic aortic aneurysms and their outcomes in this case report, it adds to the literature, outcomes, and modern management of patients with mycotic AAA with aortic graft involvement secondary to *Brucella*, especially in areas that are not endemic to *Brucella* where there is less clinical experience in treating these patients. Additionally, we share our experience treating an endograft infected by *Brucella* as endografts are becoming a primary method to treat aortic aneurysms worldwide, which will lead to an increase in the prevalence of treating infected endografts caused by all organisms.

## Notes

***Author contributions.*** Conceptualization, project administration, supervision: G. A. E. and C. S. K. Writing–original draft: B. M. M., E. L. Writing–review and editing: B. M. M., E. L., G. L. D., C. S. K., G. A. E. Visualization: B. M. M.

***Patient consent statement.*** Patient consent has been obtained, and the design of this work conforms to standards currently applied in the United States.

***Financial support.*** No financial support was utilized for the completion of this work.

***Potential conflicts of interest.*** All authors: No reported conflicts.
